# Placental, Matrilineal, and Epigenetic Mechanisms Promoting Environmentally Adaptive Development of the Mammalian Brain

**DOI:** 10.1155/2016/6827135

**Published:** 2016-03-16

**Authors:** Kevin D. Broad, Eridan Rocha-Ferreira, Mariya Hristova

**Affiliations:** Institute for Women's Health, University College London, 74 Huntley Street, London WC1N 6HX, UK

## Abstract

The evolution of intrauterine development, vivipary, and placentation in eutherian mammals has introduced new possibilities and constraints in the regulation of neural plasticity and development which promote neural function that is adaptive to the environment that a developing brain is likely to encounter in the future. A range of evolutionary adaptations associated with placentation transfers disproportionate control of this process to the matriline, a period unique in mammalian development in that there are three matrilineal genomes interacting in the same organism at the same time (maternal, foetal, and postmeiotic oocytes). The interactions between the maternal and developing foetal hypothalamus and placenta can provide a template by which a mother can transmit potentially adaptive information concerning potential future environmental conditions to the developing brain. In conjunction with genomic imprinting, it also provides a template to integrate epigenetic information from both maternal and paternal lineages. Placentation also hands ultimate control of genomic imprinting and intergenerational epigenetic information transfer to the matriline as epigenetic markers undergo erasure and reprogramming in the developing oocyte. These developments, in conjunction with an expanded neocortex, provide a unique evolutionary template by which matrilineal transfer of maternal care, resources, and culture can be used to promote brain development and infant survival.

## 1. Introduction

Two major innovations have contributed to the evolutionary success of mammals. The first is the evolution of a foetally derived placenta which enables the foetus to influence its own destiny, whilst placing a considerable burden in terms of time and energy investment on an expectant mother. Hormonal cues provided by the developing foetus influence maternal neural function, increase maternal food consumption to compensate for increased energetic costs [1], suppress oestrus and sexual behaviours [2, 3], decide the timing of parturition [4], and prime the maternal hypothalamus to initiate maternal care [5, 6]. One unique feature of placentation is that there are three matrilineal generations and hence genomes interacting in one organism at the same time (mother, foetus, and postmeiotic oocytes) which provide a mechanism by which a mother can transmit adaptive or predictive information concerning potential future environmental conditions to the developing foetal brain. It also provides a mechanism to ensure intergenerational coadaptation of the maternal hypothalamus, foetal brain, and placenta. This ensures that a foetus whose hypothalamus has coadaptively engaged with its placenta and maternal hypothalamus to provide optimal transfer of energetic resources during development will when mature have a hypothalamus that will be able to efficiently transfer energetic resources to its own offspring [7–11]. Placentation also hands disproportionate control of epigenetic inheritance to the matriline as epigenetic markers undergo global erasure and reprogramming in the developing zygote [12–14]. The evolution of placentation is also accompanied by the evolution of monoallelic gene expression or genomic imprinting, where gene expression is silenced depending on the parent of origin. Genomic imprinting is absent in egg laying mammals and only around 6 imprinted genes have been detected in a range of marsupial species (who possess a choriovitelline placenta) [15]; this is in contrast to eutherian mammals where around 150 imprinted genes have been described [15, 16]. Original theories of the evolution of imprinted genes are derived from the hypothesis that paternally and maternally expressed genes act antagonistically within a developing offspring to promote differential interests with regard to nutrient transfer, but this is difficult to reconcile with the observation that the erasure and reprogramming of epigenetic markers and imprints is under matrilineal control [12–14]. Hypotheses that explain the evolution of imprinted genes have been derived that include kinship [17], sexual antagonism [18], and varying models of maternal-offspring and placental hypothalamic coadaptation [7–11, 19]. These hypotheses may not however be mutually exclusive.

The mammalian brain has undergone profound changes during its evolution. The huge expansion of the neocortex especially in primates has facilitated a shift in the complexity of social interactions and in primates complex social cues replace changes in hormonal states to promote maternal and sexual responses. Parturition is not required to initiate maternal behaviour which is therefore displayed towards a range of offspring in a social group and sexual interactions may occur out of oestrus and are used to reinforce social attachment. The most important phases of neocortical development and socialisation occur during periods of maternal attachment, among stable groups of females that extend over more than one generation. There are often complex social hierarchies within these groups and social rank is often inherited matrilineally from the mother [20].

The developing mammalian brain can demonstrate remarkable plastic and adaptive responses many of which serve to adapt the brain to an environment that it will encounter when mature. This may account for the ease in which mammals have undergone adaptive radiation into a wide range of environmental niches, where evolution of the brain and neuronal phenotype has paralleled adaptations in physical structure. In this review we focus on the importance of a number of mechanisms that contribute to this and emphasise the importance of the matriline in these mechanistic processes. We also suggest that failure in these processes can result in the induction of deleterious neural and peripheral phenotypes that cannot be viewed as traditionally pathological but are phenotypes which are adaptive to the environmental conditions with which an individual or its mother interacted during early development. Subsequent shifts in environmental conditions during an organism's later development may leave it with a poorly environmentally adaptive phenotype and a propensity for health problems.

## 2. Predictive Adaptive Responses

The hypothesis of predictive adaptive responses or plasticity (PAR) was originally developed at a whole organism level to provide a mechanistic framework by which a developing organism can attempt to predict and modify its developmental phenotype to accurately match the environment that it will encounter when mature. It also incorporated a range of ideas that attempted to explain why early life experiences are associated with an increased risk of disease in later life, especially in contemporary human societies [21–23]. There are four implications of the predictive adaptive response hypothesis. Firstly, the early life environment is predictive of the adult environment. This is likely to be a more reliable proposition in animal species with short intergenerational times (mice 4-5 weeks; cf. humans 13–15 years), with the exception of humans where technological advances and cultural transmission have produced extended environmental stability. Secondly, an organism which has during development successfully predicted its adult environment will benefit from increases in both survival and Darwinian fitness. Thirdly, successful predictive responses are primarily advantageous to the organism until reproductive age, and for reproduction itself, postreproductive beneficial or deleterious effects on an organism's health do not affect Darwinian fitness. And finally this predictive mechanism may incorporate a fatal evolutionary flaw as a mismatch between an environmental prediction and subsequent reality may lead to a poorly adaptive phenotype and health problems [20–22].

A range of predictive adaptive responses involving the brain have been described in a number of mammalian species. One of the simplest and earliest related to the observation that vole pups (*Microtus pennsylvanicus*) born in late summer and early autumn have much thicker coats than those born in the spring. The proximal cue for this is a reduction in day length, which is transmitted to the developing vole* in utero* via melatonin signalling originating in the mother [24, 25]. It is a tautological prediction that reduced day length precedes winter and this adaptation enables autumn born voles to survive winter better than they would with thinner coats. Generally these predictive adaptive responses are advantageous to survival but in dynamically changing environments especially when intergenerational times are extended, predictive adaptive responses may have contingent or dichotomous properties which may lead to a mismatch between phenotype and environment. One of the most studied examples is the relationship between maternal nutrition and offspring metabolism. A range of epidemiological observations has established that maternal nutrition developmentally programmes the metabolic, behavioural, and developmental phenotype of their offspring. Well-nourished mothers have offspring who are adapted to affluent environments; offspring born to mothers with restricted nutrition are adapted to impoverished environments. Children born with low birthweights (which is thought to reflect impoverished intrauterine nutritional supply) enter puberty early [26–29] and have a preference for high fat foods [30, 31] and higher satiation thresholds [32] and a smaller stature [33], phenotypical characteristics that are adaptive in a life of impoverished circumstances. The proof of principle of this adaptive response has been characterised in a range of animal species, as direct human evidence is scant and incorporates a range of confounders derived from adult socioeconomic status [34]. Anecdotal evidence obtained from concentration and prisoner-of-war camps suggests that physically large individuals died first whilst at least some small individuals survived [35]. And in a famine afflicted Ethiopian population a high birthweight was associated with a 9-fold greater risk of rickets [36]. Epidemiological studies have however provided inconclusive evidence. Between 1887 and 1888, harvests in Finland failed and poor people suffered greatly in terms of both adult and infant mortality. A key prediction of the predictive adaptive response hypothesis should be that poor individuals who were born in impoverished years should be more resistant to the effects of the famine than those born in more affluent years; however this was not the case and poor people who were born in more affluent years were less affected than those born in impoverished years [34]. Gluckman and Hanson argued that the poor physical and economic conditions of those born in impoverished years may have persisted throughout life and therefore their less resilient responses did not represent a predictive adaptive response to the 1887-8 famine [22].

The predictive adaptive response hypothesis has been used to explain why variations in the early interuterine and neonatal environment exert profound effects on health and disease in later life. A mismatch between an environmental prediction made during early development and subsequent reality may mean that an organism may have a poorly adapted phenotype to its adult environment which may predispose it to health problems. Babies reared in affluent environments enjoy a reduced risk of a range of diseases as adults including diabetes [37–39]; however babies born in impoverished but who move to more affluent environments demonstrate increased rates of metabolic and cardiovascular diseases in later life [40, 41].

### 2.1. The Evolution of Placentation Facilitates Intergenerational Adaptive Neural Plasticity, Information Transfer, and Coadaptation

Placentation is universally thought to be an iconic feature of mammalian evolution but this view is incomplete and based on observations of extant mammalian species. For much of early mammalian evolution the majority of mammal species despite possessing a suite of iconic mammalian features including hair, thermoregulation, lactation, and a highly developed neocortex laid eggs. The subsequent evolution of placentation ensured that during gestation there are three matrilineal generations, present in one organism at one time (mother, foetus, and postmeiotic oocytes) which provides a unique template by which a mother can transmit environmental information to her developing foetus and her potential grandchildren. The placenta is produced by the developing foetus and provides a mechanism by which it can attempt to control the physiology and neural function of its mother to its own advantage. Progesterone secreted by the placenta suppresses sexual activity [2, 3]; its metabolite allopregnanolone increases maternal food intake [1], exerts a neuroprotective influence [42], and promotes hippocampal neurogenesis in both foetus and mother [43].

Placental hormones also prime the oxytocin system in anticipation of parturition where oxytocin release is required to support uterine contractions, milk ejection, and maternal behaviour in the mother [5, 6] and to promote solicitation from and attachment to the mother in the offspring [44], this acts to coordinate the evolutionary appropriate behavioural responses in both mother and infant. One potential caveat of placentation means that the mother has to contend with the potential immune response generated by an invasion of semiallogenic foetal trophoblast cells [45]. This has facilitated the evolution of a range of coadaptive responses; the foetal placenta has adapted to avoid the maternal immune system but maternal natural killer cells have also adapted to activate growth and development of the placenta, by promoting angiogenesis, vascular remodelling, and trophoblast invasion of the uterus [46].

The developing foetus begins to exert considerable leverage on the neural function of its maternal hypothalamus during early placental development at the same time as it develops a hypothalamus of its own [7–11, 47, 48]. This intimate transgenerational interaction of two separate genomes and hypothalami can provide an efficient mechanism to promote both information transfer about environmental cues and coadaptive function across generations. This model of development has been termed hypothalamic/placental coadaptation and states that a developing foetus whose placenta and hypothalamus are coadapted to regulate optimal resource transfer from the mother will develop an adult hypothalamus that will be able to efficiently transfer resources to its own offspring* in utero* via placentation or postnatally via lactation and efficient maternal care [7–11, 47, 48].

### 2.2. The Evolution of Genomic Imprinting

Genomic imprinting refers to a form of monoallelic gene expression whereby the expression of an allele depends on its parental origin. The fact that genomic imprinting is absent in egg laying mammals and the fact that there are only 6 or so recorded imprinted genes in marsupials but around 150 imprinted genes in eutherian mammals have driven the development of over 14 interesting theories that explain their existence [14, 48]. Most of these theories are difficult to test empirically and do have intellectual limitations. A number of these theories may not be mutually exclusive. In the context of this review two similar hypotheses involving transgenerational coadaptation and information transfer provide interesting explanations for the evolution of genomic imprinting. Wolf and Hager, 2006 [19], suggested that monoallelically maternally expressed genes facilitate coadaptation of interacting maternal and offspring traits during development, which acts to increase offspring fitness. One prediction of this is that increasingly biased patterns of monoallelic maternal gene expression would increase the overall fitness of any subsequent generation. Keverne argued that imprinted genes facilitate the coadaptation of the developing foetal hypothalamus and placenta with the hypothalamus of the mother [7–11, 47, 48]. This would also increase overall fitness of offspring by promoting the transgenerational formation of a hypothalamic centred network that acts to adapt and stabilise optimal energetic and nutrient transfer between an expectant mother and the developing foetal brain and placenta. The hypothalamic neural circuitry responsible for the extraction of maternal energetic resources displays a remarkable overlap with the hypothalamic circuitry responsible for the provision of resources to offspring. This ensures that a brain adapted to extract optimal energetic resources when developing will be predisposed to provide optimal energetic resources to its own young.

Many imprinted genes are coexpressed in the developing hypothalamus and placenta, and a range of studies using mice with an inactivated paternally expressed gene 3 (*Peg3*) has provided a range of evidence that supports the role of imprinted genes in the development of coadapted responses between the hypothalamus and placenta. The* Peg3* gene can be inactivated in the maternal hypothalamus but not the placenta or hypothalamus of offspring or be functional in the maternal hypothalamus but inactivated in the hypothalamus or placenta of offspring. Both of these paradigms produce remarkably similar deficits in hypothalamic cell number, maternal behaviour, and impaired suckling, which leads to impaired growth, onset of puberty, and reproduction [48, 49].

Disruption of this hypothalamic-placental response with 24 h of maternal food deprivation induces a significant decrease in* Peg3* expression in the placenta and other gene expression changes that are induced resemble those when the* Peg3* gene is inactivated and include the disruption of genes expression linked to autophagy and ribosomal turnover. In the foetal hypothalamus* Peg3* expression is increased following 24 h of maternal food deprivation. These results suggest that the foetus attempts to control its own destiny during periods of acute starvation by a short term sacrifice of its placenta which preserves resources required for neuronal development [48, 50].

Imprinted genes are found in clusters around heritable imprint control regions (ICRs), which are thought to be important for the regulation of monoallelic gene expression and disruption to these can be associated with Prader-Willi and Angelman's syndrome [51]. Imprinted control regions are highly conserved and predominately matrilineal and have important roles for embryo survival and maternal/foetal interactions during development [52, 53]. They are also thought to have important roles in the allocation of matrilineal time and energy, which is an important evolutionary consideration given that often 90% of mammalian adult female life is committed to pregnancy, lactation, and maternal care.

### 2.3. Epigenetic Considerations

The development of the brain is by definition a complex epigenetic process; neural systems are designed to respond to environmental changes as the interactions of over a trillion synaptic connections both are activity dependent and can be subjected to epigenetic modification. This occurs throughout the life of an individual but at key stages of development, epigenetic modification of neural function is under disproportionate control of the matriline. The erasure and reprogramming of the epigenetic landscape in the developing oocyte, interaction of the developing brain and placenta with environmental cues, the ability of a developing brain to accurately assess present conditions and accurately anticipate the future, and programming of the postpartum brain via efficient maternal care and lactation are all disproportionately matrilineal processes. No two brains are ever alike and even monozygotic twins exhibit increasing differences in behaviour and predisposition to psychiatric disorders as they age [54, 55] which are thought to be associated with subtle epigenetic influences resulting from minor variations in developmental environments. Immediately before birth, coordinated epigenetic and transcriptional remodelling begins to facilitate the brains adaptation to an extrauterine environment. At birth the foetus is disconnected from its own placenta and supply of placental hormones controlled by its own genome, including allopregnanolone, which facilitates a rapid shift in GABA function [46, 47]. These processes enable rapid postnatal synaptogenesis and neocortical expansion which allows the integration of activity dependent neuronal gene expression and development with salient environmental and developmental cues. In humans this remodelling extends from just before birth to the first year of life [56, 57]. Activity dependent neuronal gene expression, remodelling, and maturation during development are critical for the proper development of neural circuits. In the visual, auditory, and attentional systems, disruption of this process results in a delay in the maturation of the systems supporting these functions and lifelong cognitive deficits [58, 59]. This underappreciated process has important implications for neonatal medicine, where interventions during this critical transformative process may induce lifelong deficits in neuronal function [60, 61]. A range of intergenerational based matrilineal epigenetic influences on neural function and behaviour have been described mainly in the context of maternal behaviour and stress responses [62–64]. Rodents reared by mothers who display poor quality maternal care acquire heritable epigenetic changes to their oestrogen and oxytocin receptor systems and then in turn display poor quality maternal care to their own offspring [62, 63]. Although both developmental and intergenerational epigenetic modifications are under disproportionate control of the matriline, paternal transgenerational influences in neural function are also important. Male mice conditioned to an aversive stimulus paired with an odour (acetophenone) which activates a specific odorant receptor (Olfr151) have offspring and grandoffspring that demonstrate enhanced biological sensitivity to acetophenone and increases in Olfr151 activity. Bisulfite sequencing of sperm DNA in acetophenone conditioned fathers and their offspring demonstrated CpG hypomethylation in the Olfr151 gene. This paternal transgenerational mechanism is adaptive in that it allows potential predator avoidance cues to be transferred quickly through a population [64]. Other examples of paternal epigenetic influence may not be adaptive and there is evidence that paternal age, nutrition, substance abuse, and stress may predict mental and physical health in offspring [65–68]. The exact molecular mechanisms by which paternal epigenetic influences can be transmitted across generations are poorly understood but are thought to require the active cooperation of the matriline, as DNA methylation marks are globally erased and reprogrammed in the developing zygote following fertilisation [12–14]. Mothers may also modify paternal influences on neural development by dynamically adjusting their reproductive investment in response to the qualities of their mate. Female mice that mate with males housed in a socially enriched environment exhibit increased levels of maternal care towards their offspring of pup nursing and licking towards their offspring, which are associated with increases in expression of brain derived neurotrophic factor in the maternal hypothalamus [68].

The evolution of paternally derived intergenerational effects on offspring fitness and phenotype may suggest one further mechanism by which adaptive traits can be passed rapidly through an expanding population. Beneficial genetic variations and epigenetic modifications of monoallelically paternally expressed genes will spread far more rapidly through a population than those of monoallelically expressed maternally expressed genes. This is because mammals typically have fewer ancestral fathers than mothers as in males freed from the energetic burden of placentation reproductive success is based on competing to impregnate as many females as possible. This means that many low status males fail to breed and yet high status males often have large numbers of offspring. In populations that are moving into novel environments, who then subsequently undergo a rapid expansion this reproductive asymmetry coupled with the evolution of paternally expressed (and hence maternally silenced) gene expression provides an elegant mechanism to rapidly distribute traits that are adaptive to neural function. Adaptive gene variants and epigenetic modifications of paternally expressed genes will spread extremely rapidly in such rapidly expanding population, especially in circumstances where there are few ancestral or founding fathers. This is an important adaptive trait when populations are moving into new environmental niches. There are animal examples of this gender asymmetry of ancestral influences but one rare but illustrative human example of this process relates to the amazing fecundity of Genghis Khan who was thought to have sired over 1,500 children. This productivity was also replicated by his sons and grandsons. As a consequence there are now over 16 million men in Asia who can trace their Y chromosome lineage to an extremely small number of reproductively successful men living in the 12 and 13th centuries [69].

## 3. Discussion

In circumstances where an organism's early life environment provides accurate predictive cues to the environmental conditions prevailing when adult, the ability of the developing foetal and neonatal mammalian brain to adjust its development to optimally match its function to the requirements of its adult environment confers profound advantages in terms of survival and reproductive fitness. One evolutionary development that elegantly facilitates this process in eutherian mammals is the development of a foetally derived placenta, by which the foetus can attempt to control its own destiny and hormonally regulate the maternal hypothalamus to leverage maternal resources for its own benefit. This is a unique situation that requires the interaction and coadaptation of three matrilineal generations and hence genomes interacting in one organism at the same time (mother, foetus, and postmeiotic oocytes). This provides a template by which a mother can transmit adaptive information concerning present and potential future environmental conditions to the developing foetal brain over an extended period. When combined with the evolution of imprinted genes and disproportionate maternal control over the mechanisms of epigenetic inheritance, this provides a mechanism to ensure intergenerational coadaptation of the maternal hypothalamus, developing brain, and placenta, integrate potential maternal and paternal interests, and promote the optimal allocation of maternal resources and transfer of matrilineal cultural influences. In circumstances either where the early environment provides inaccurate cues to the environmental conditions prevailing when adult due to rapid environmental change or when disruptions to normal neural development occur, the mismatch between the environmental predictions made during early development and subsequent reality may mean that an organism may have a poorly adapted phenotype to its adult environment which may predispose it to health problems. An appreciation of these underlying evolutionary salient processes may provide a novel perspective on the casual mechanisms of a range of health problems. The concept of a brain that is not pathological in the classical sense but it is simply mismatched to its environment has been most extensively studied in the context of ancestral and early developmental nutrition [21–23, 26–28, 31–35, 40, 41]. However, this concept can be extended to provide insights into the development of a range of alternative neural phenotypes. These include psychopathy whose defining characteristics of impulsivity, recklessness, lack of empathy, and a predisposition to violence are adaptive in extreme conflict driven environments but catastrophically maladaptive in normal circumstances [70, 71]. Examination of the adaptive potential of a range of neural and cognitive deficits in the context of evolutionary derived foetocentric brain and placental development, epigenetics and environmental adaptation may provide novel insights into the development and potential treatment of a range of health, neurological, and cognitive disorders.

## References

[1] Fudge M. A., Kavaliers M., Ossenkopp K.-P. (2006). Allopregnanolone produces hyperphagia by reducing neophobia without altering food palatability. *European Neuropsychopharmacology*.

[2] Asa C. S., Goldfoot D. A., Garcia M. C., Ginther O. J. (1984). The effect of estradiol and progesterone on the sexual behavior of ovariectomized mares. *Physiology & Behavior*.

[3] Graham M. D., Gardner Gregory J., Hussain D., Brake W. G., Pfaus J. G. (2015). Ovarian steroids alter dopamine receptor populations in the medial preoptic area of female rats: implications for sexual motivation, desire, and behaviour. *European Journal of Neuroscience*.

[4] Ng P. C. (2000). The fetal and neonatal hypothalamic-pituitary-adrenal axis. *Archives of Disease in Childhood: Fetal and Neonatal Edition*.

[5] Broad K. D., Lévy F., Evans G., Kimura T., Keverne E. B., Kendrick K. M. (1999). Previous maternal experience potentiates the effect of parturition on oxytocin receptor mRNA expression in the paraventricular nucleus. *European Journal of Neuroscience*.

[6] Broad K. D., Curley J. P., Keverne E. B. (2006). Mother-infant bonding and the evolution of mammalian social relationships. *Philosophical Transactions of the Royal Society B: Biological Sciences*.

[7] Keverne E. B., Curley J. P. (2008). Epigenetics, brain evolution and behaviour. *Frontiers in Neuroendocrinology*.

[8] Keverne E. B. (2014). Mammalian viviparity: a complex niche in the evolution of genomic imprinting. *Heredity*.

[9] Keverne E. B. (2014). Significance of epigenetics for understanding brain development, brain evolution and behaviour. *Neuroscience*.

[10] Keverne E. B., Pfaff D. W., Tabansky I. (2015). Epigenetic changes in the developing brain: effects on behavior. *Proceedings of the National Academy of Sciences of the United States of America*.

[11] Keverne E. B. (2015). Genomic imprinting, action, and interaction of maternal and fetal genomes. *Proceedings of the National Academy of Sciences of the United States of America*.

[12] Guo H., Zhu P., Yan L., Li R., Hu B., Lian Y. (2014). The DNA methylisation landscape of human early human embryos. *Nature*.

[13] Smith Z. D., Chan M. M., Humm K. C. (2014). DNA methylation dynamics of the human preimplantation embryo. *Nature*.

[14] Seisenberger S., Peat J. R., Hore T. A., Santos F., Dean W., Reik W. (2013). Reprogramming DNA methylation in the mammalian life cycle: building and breaking epigenetic barriers. *Philosophical Transactions of the Royal Society B*.

[15] Renfree M. B., Suzuki S., Kaneko-Ishino T. (2013). The origin and evolution of genomic imprinting and viviparity in mammals. *Philosophical Transactions of the Royal Society B: Biological Sciences*.

[16] Williamson C. M., Blake A., Thomas S. (2013). *Mouse Imprinting Data and References*.

[17] Haig D. (2000). The kinship theory of genomic imprinting. *Annual Review of Ecology and Systematics*.

[18] Day T., Bonduriansky R. (2004). Intralocus sexual conflict can drive the evolution of genomic imprinting. *Genetics*.

[19] Wolf J. B., Hager R. (2006). A maternal-offspring coadaptation theory for the evolution of genomic imprinting. *PLoS Biology*.

[20] Dunbar R. I. (1983). Reproductive biology of the great apes: comparative and biomedical perspectives: C.E. Graham (Editor). Academic Press, New York, 1981, xviii + 437 pp. US $ 48.50, ISBN 0-12-295020-8. hardback. *Behavioural Processes*.

[21] Bateson P., Barker D., Clutton-Brock T. (2004). Developmental plasticity and human health. *Nature*.

[22] Gluckman P., Hanson M. (2005). *The Fetal Matrix: Evolution, Development and Disease*.

[23] Gluckman P. D., Hanson M. A., Bateson P. (2009). Towards a new developmental synthesis: adaptive developmental plasticity and human disease. *The Lancet*.

[24] Lee T. M., Zucker I. (1988). Vole infant development is influenced perinatally by maternal photoperiodic history. *American Journal of Physiology—Regulatory Integrative and Comparative Physiology*.

[25] Lee T. M., Spears N., Tuthill C. R., Zucker I. (1989). Maternal melatonin treatment influences rates of neonatal development of meadow vole pups. *Biology of Reproduction*.

[26] Adair L. S. (2001). Size at birth predicts age at menarche. *Pediatrics*.

[27] Sloboda D. M., Hart R., Doherty D. A., Pennell C. E., Hickey M. (2007). Rapid communication—age at menarche: influences of prenatal and postnatal growth. *The Journal of Clinical Endocrinology & Metabolism*.

[28] Nettle D., Coall D. A., Dickins T. E. (2010). Birthweight and paternal involvement predict early reproduction in British women: evidence from the national child development study. *American Journal of Human Biology*.

[29] Cooper C., Kuh D., Egger P., Wadsworth M., Barker D. (1996). Childhood growth and age at menarche. *British Journal of Obstetrics and Gynaecology*.

[30] Perälä M.-M., Männistö S., Kaartinen N. E. (2012). Body size at birth is associated with food and nutrient intake in adulthood. *PLoS ONE*.

[31] Doornweerd S., IJzerman R. G., Weijs P. J., Diamant M., de Geus E. J., Boomsma D. I. (2015). Lower birth weight is associated with alterations in dietary intake in adolescents independent of genetic factors: a twin study. *Clinical Nutrition*.

[32] Ross M. G., Desai M. (2014). Developmental programming of appetite/satiety. *Annals of Nutrition and Metabolism*.

[33] Lindberg J., Norman M., Westrup B., Öhrman T., Domellöf M., Berglund S. K. (2015). Overweight, obesity, and body composition in 3.5- and 7-year-old Swedish Children born with marginally low birth weight. *Journal of Pediatrics*.

[34] Hayward A. D., Rickard I. J., Lummaa V. (2013). Influence of early-life nutrition on mortality and reproductive success during a subsequent famine in a preindustrial population. *Proceedings of the National Academy of Sciences of the United States of America*.

[35] Bateson P. (2001). Fetal experience and good adult design. *International Journal of Epidemiology*.

[36] Chali D., Enquselassie F., Gesese M. (1998). A case control study on determinants of rickets. *Ethiopian Medical Journal*.

[37] Barker D. J. P. (2001). *Fetal Origins of Cardiovascular and Lung Disease*.

[38] Barker D. J. P., Forsén T., Uutela A., Osmond C., Eriksson J. G. (2001). Size at birth and resilience to effects of poor living conditions in adult life: longitudinal study. *British Medical Journal*.

[39] Eriksson J. G., Osmond C., Lindi V. (2003). Interactions between peroxisome proliferator-activated receptor gene polymorphism and birth length influence risk for type 2 diabetes. *Diabetes Care*.

[40] Patel J. V., Vyas A., Cruickshank J. K. (2006). Impact of migration on coronary heart disease risk factors: comparison of Gujaratis in Britain and their contemporaries in villages of origin in India. *Atherosclerosis*.

[41] Ebrahim S., Kinra S., Bowen L. (2010). The effect of rural-to-urban migration on obesity and diabetes in India: a cross-sectional study. *PLoS Medicine*.

[42] Knight S. R., Davidson C., Young A. M. J., Gibson C. L. (2012). Allopregnanolone protects against dopamine-induced striatal damage after in vitro ischaemia via interaction at GABA A receptors. *Journal of Neuroendocrinology*.

[43] Quadrato G., Elnaggar M. Y., Duman C., Sabino A., Forsberg K., Di Giovanni S. (2014). Modulation of GABAA receptor signaling increases neurogenesis and suppresses anxiety through NFATc4. *The Journal of Neuroscience*.

[44] Simpson E. A., Sclafani V., Paukner A. (2014). Inhaled oxytocin increases positive social behaviors in newborn macaques. *Proceedings of the National Academy of Sciences of the United States of America*.

[45] Rossant J., Cross J. C. (2001). Placental development: lessons from mouse mutants. *Nature Reviews Genetics*.

[46] Moffett A., Loke C. (2006). Immunology of placentation in eutherian mammals. *Nature Reviews Immunology*.

[47] Keverne E. B. (2012). Importance of the matriline for genomic imprinting, brain development and behaviour. *Philosophical Transactions of the Royal Society of London B, Biological Sciences*.

[48] Broad K. D., Keverne E. B. (2011). Placental protection of the fetal brain during short-term food deprivation. *Proceedings of the National Academy of Sciences of the United States of America*.

[49] Curley J. P., Pinnock S. B., Dickson S. L. (2005). Increased body fat in mice with a targeted mutation of the paternally expressed imprinted gene Peg3. *The FASEB Journal*.

[50] Zeltser L. M., Leibel R. L. (2011). Roles of the placenta in fetal brain development. *Proceedings of the National Academy of Sciences of the United States of America*.

[51] Rabinovitz S., Kaufman Y., Ludwig G., Razin A., Shemer R. (2012). Mechanisms of activation of the paternally expressed genes by the Prader-Willi imprinting center in the Prader-Willi/Angelman syndromes domains. *Proceedings of the National Academy of Sciences of the United States of America*.

[52] Hutter B., Bieg M., Helms V., Paulsen M. (2010). Imprinted genes show unique patterns of sequence conservation. *BMC Genomics*.

[53] O'Connell M. J., Loughran N. B., Walsh T. A., Donoghue M. T. A., Schmid K. J., Spillane C. (2010). A phylogenetic approach to test for evidence of parental conflict or gene duplications associated with protein-encoding imprinted orthologous genes in placental mammals. *Mammalian Genome*.

[54] Kordi-Tamandani D. M., Sahranavard R., Torkamanzehi A. (2013). Analysis of association between dopamine receptor genes' methylation and their expression profile with the risk of schizophrenia. *Psychiatric Genetics*.

[55] Mezuk B., Heh V., Prom-Wormley E., Kendler K. S., Pedersen N. L. (2015). Association between major depression and type 2 diabetes in midlife: findings from the Screening Across the Lifespan Twin Study. *Psychosomatic Medicine*.

[56] Siegmund K. D., Connor C. M., Campan M. (2007). DNA methylation in the human cerebral cortex is dynamically regulated throughout the life span and involves differentiated neurons. *PLoS ONE*.

[57] Shulha H. P., Cheung I., Guo Y., Akbarian S., Weng Z. (2013). Coordinated cell type-specific epigenetic remodeling in prefrontal cortex begins before birth and continues into early adulthood. *PLoS Genetics*.

[58] Gordon K. A., Wong D. D. E., Valero J., Jewell S. F., Yoo P., Papsin B. C. (2011). Use it or lose it? Lessons learned from the developing brains of children who are deaf and use cochlear implants to hear. *Brain Topography*.

[59] Tropea D., Van Wart A., Sur M. (2009). Molecular mechanisms of experience-dependent plasticity in visual cortex. *Philosophical Transactions of the Royal Society B: Biological Sciences*.

[60] Ter Wolbeek M., Kavelaars A., de Vries W. B. (2015). Neonatal glucocorticoid treatment: long-term effects on the hypothalamus-pituitary-adrenal axis, immune system, and problem behavior in 14–17 year old adolescents. *Brain, Behavior, and Immunity*.

[61] Saavedra-Rodríguez L., Feig L. A. (2013). Chronic social instability induces anxiety and defective social interactions across generations. *Biological Psychiatry*.

[62] Monk C., Spicer J., Champagne F. A. (2012). Linking prenatal maternal adversity to developmental outcomes in infants: the role of epigenetic pathways. *Development and Psychopathology*.

[63] Stolzenberg D. S., Champagne F. A. (2015). Hormonal and non-hormonal bases of maternal behavior: the role of experience and epigenetic mechanisms. *Hormones and Behavior*.

[64] Dias B. G., Ressler K. J. (2014). Parental olfactory experience influences behavior and neural structure in subsequent generations. *Nature Neuroscience*.

[65] Essex M. J., Thomas Boyce W., Hertzman C. (2013). Epigenetic vestiges of early developmental adversity: childhood stress exposure and dna methylation in adolescence. *Child Development*.

[66] Pembrey M. E., Bygren L. O., Kaati G. (2006). Sex-specific, male-line transgenerational responses in humans. *European Journal of Human Genetics*.

[67] Kaati G., Bygren L. O., Pembrey M., Sjöström M. (2007). Transgenerational response to nutrition, early life circumstances and longevity. *European Journal of Human Genetics*.

[68] Mashoodh R., Franks B., Curley J. P., Champagne F. A. (2012). Paternal social enrichment effects on maternal behavior and offspring growth. *Proceedings of the National Academy of Sciences of the United States of America*.

[69] Zerjal T., Xue Y., Bertorelle G. (2003). The genetic legacy of the Mongols. *American Journal of Human Genetics*.

[70] Glenn A. L., Raine A. (2009). Psychopathy and instrumental aggression: evolutionary, neurobiological, and legal perspectives. *International Journal of Law and Psychiatry*.

[71] Crawford C., Salmon C. (2002). Psychopathology or adaptation? Genetic and evolutionary perspectives on individual differences and psychopathology. *Neuro Endocrinology Letters*.

